# Review on PACAP-Induced Transcriptomic and Proteomic Changes in Neuronal Development and Repair

**DOI:** 10.3390/ijms19041020

**Published:** 2018-03-29

**Authors:** Adam Rivnyak, Peter Kiss, Andrea Tamas, Dorottya Balogh, Dora Reglodi

**Affiliations:** Department of Anatomy, MTA-PTE PACAP Research Team, Neuroscience Centre, University of Pecs Medical School, 7624 Pécs, Hungary; rivnyakadam@gmail.com (A.R.); peter.kiss@aok.pte.hu (P.K.); andreatamassz@gmail.com (A.T.); baloghdorottya333@gmail.com (D.B.)

**Keywords:** PACAP, neuropeptide, development, neurotrophic, neuroprotective

## Abstract

Pituitary adenylate cyclase activating polypeptide (PACAP) is a neuropeptide with widespread occurrence and diverse biological effects. Among its several different effects, of special importance is the action of PACAP on neuronal proliferation, differentiation and migration, and neuroprotection. The neuroprotective mechanism of PACAP is both direct and indirect, via neuronal and non-neuronal cells. Several research groups have performed transcriptomic and proteomic analysis on PACAP-mediated genes and proteins. Hundreds of proteins have been described as being involved in the PACAP-mediated neuroprotection. In the present review we summarize the few currently available transcriptomic data potentially leading to the proteomic changes in neuronal development and protection. Proteomic studies focusing on the neuroprotective role of PACAP are also reviewed and discussed in light of the most intriguing and promising effect of this neuropeptide, which may possibly have future therapeutic potential.

## 1. General Overview

Pituitary adenylate cyclase activating polypeptide (PACAP) is a neuropeptide with widespread occurrence and diverse biological effects [1,2]. PACAP occurs at highest concentrations in the nervous system, where it functions as a neuromodulatory and neurotransmitter peptide. In the nervous system, PACAP acts, amongst others, on hypothalamic hormone release and hypothalamo-hypophyseal hormonal regulatory pathways [3,4], on central thermoregulatory and feeding routes [5,6,7,8], on neurogenesis [9] and on cognitive performance [10,11,12]. Of special importance is the action of PACAP on neuronal proliferation, differentiation and migration, whereby the peptide has significant effects in the development of the nervous system [13,14]. Several factors that are important during development are upregulated after injuries. Mechanisms driven by these factors are re-employed in post-injury recovery and regeneration [15,16]. In this regard, PACAP is also a neurotrophic factor that is upregulated in several types of neuronal pathological conditions [16]. This has been shown following nerve transection, neuronal inflammations, stroke and traumatic brain injury [16,17,18]. The protective action of endogenous PACAP is also supported by the increased vulnerability to various harmful stressors of mice lacking PACAP (PACAP gene knockout mice) [19]. A more severe injury has been described in PACAP knockout mice in models of cerebral ischemia [20], in spinal cord traumatic injury [21] and in retinal ischemia [22]. Not surprisingly, exogenous PACAP is a very potent neuroprotective agent, as proven by dozens of neuronal injury models, both in vitro and in vivo [16,17,18,23,24,25].

The neuroprotective mechanism of PACAP is both direct and indirect, via neuronal and non-neuronal cells [14,18]. PACAP acts through its specific G protein-coupled PAC1 receptor, which only binds PACAP, and through the VPAC1 and VPAC2 receptors, which also bind the structurally related vasoactive intestinal peptide (VIP) with similar affinity [26,27]. The neuroprotective signaling is mediated mainly via the PACAP-specific PAC1 receptor [24,28], through which anti-apoptotic and regeneration-promoting effects are mediated. This also explains why the closely related VIP acting on VPAC1/2 receptors exerts less potent neuroprotective effects, while other members of the peptide family (secretin, glucagon) have no neuroprotective efficacy [17,25,26,28]. However, the involvement of VPAC receptors also seems to be important, mainly conveying anti-inflammatory actions of the peptide. Via receptorial mechanisms, PACAP stimulates cAMP/PKA- (cyclic adenosine monophosphate/protein kinase A) mediated signaling, and also PI3K-pathways (phosphoinositol 3 kinase) and calcium-regulated mechanisms [27,29]. In addition, PACAP transactivates growth factor-related pathways [30] and penetrates the cell membrane directly, through receptor-independent uptake [31]. By these pathways, PACAP regulates a network of signaling molecules involved in neuronal protection and repair.

Several research groups have performed transcriptomic and proteomic analysis on PACAP-mediated genes and proteins. Hundreds of proteins have been described as involved in the PACAP-mediated neuroprotection [17,18,24,25]. The aim of the present review is to summarize the limited transcriptomic data currently available which could potentially lead to the proteomic changes in neuronal development and protection. Proteomic studies focusing on the neuroprotective effect of PACAP will also be reviewed and discussed in light of the most intriguing and promising effect of this neuropeptide.

**Figure 1:**
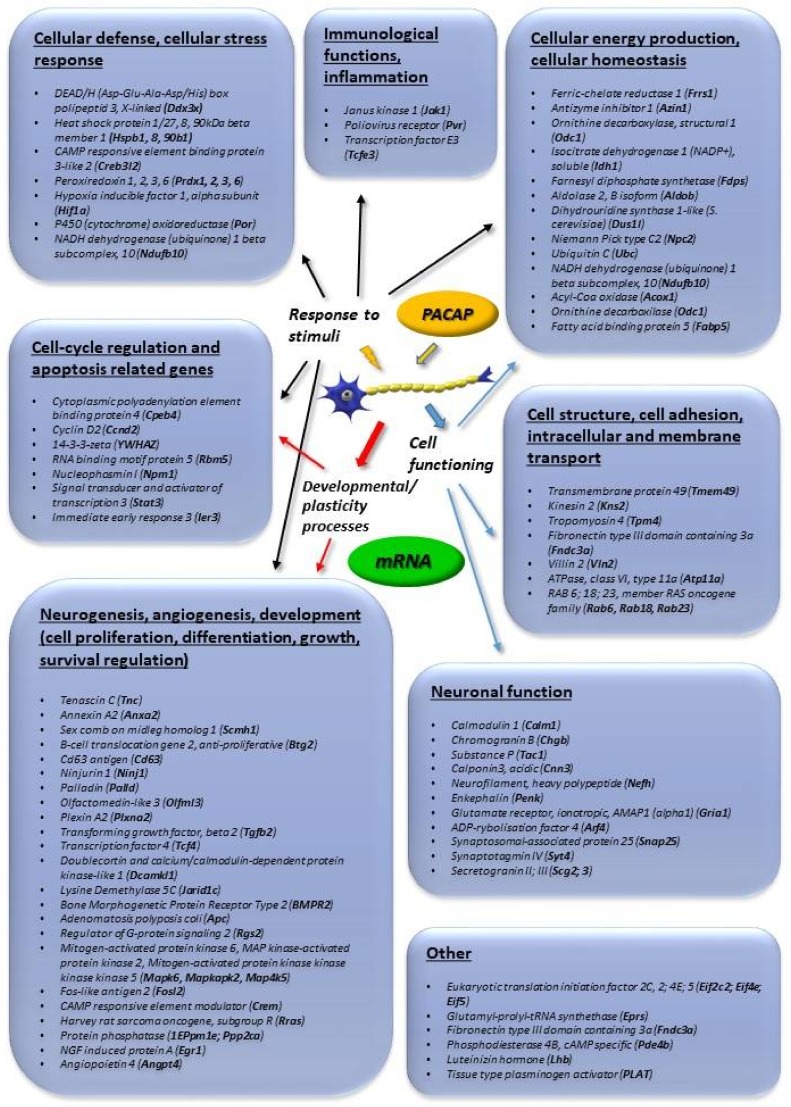
Main in vitro transcriptomic changes induced by pituitary adenylate cyclase activating polypeptide (PACAP). Genes upregulated more than 2-fold are listed. The data in the figure come from the references: [32,33,34,35,46].

**Figure 2:**
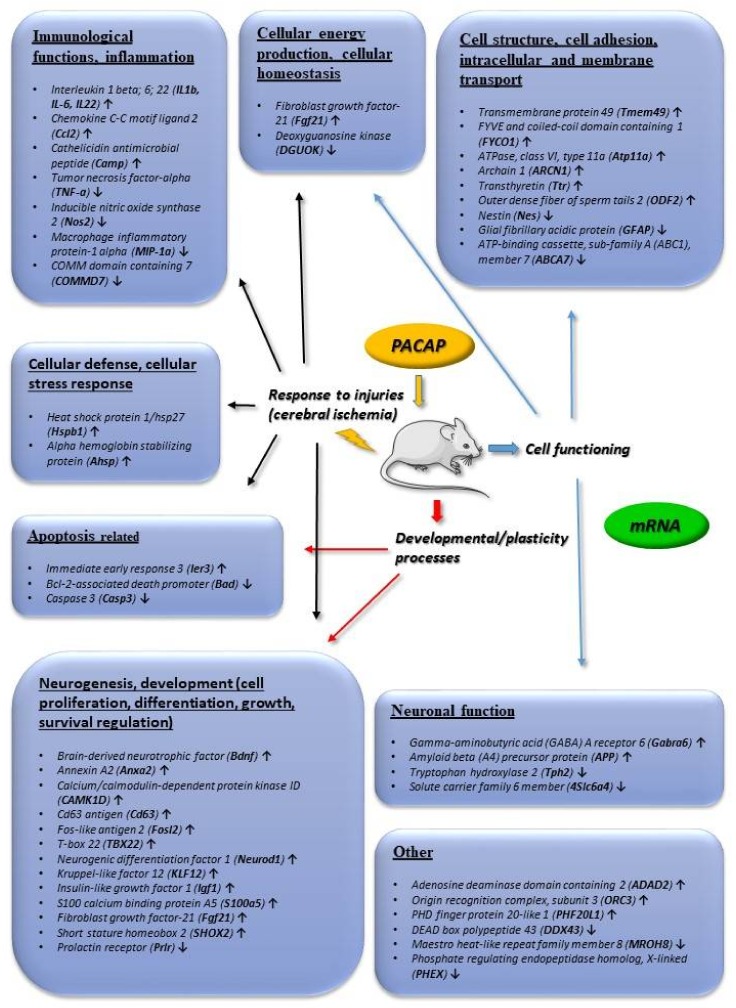
Main in vivo transcriptomic changes induced by PACAP. Arrows indicate main up or downregulated genes. The data in the figure come from the references: [32,33,36,37].

**Figure 3:**
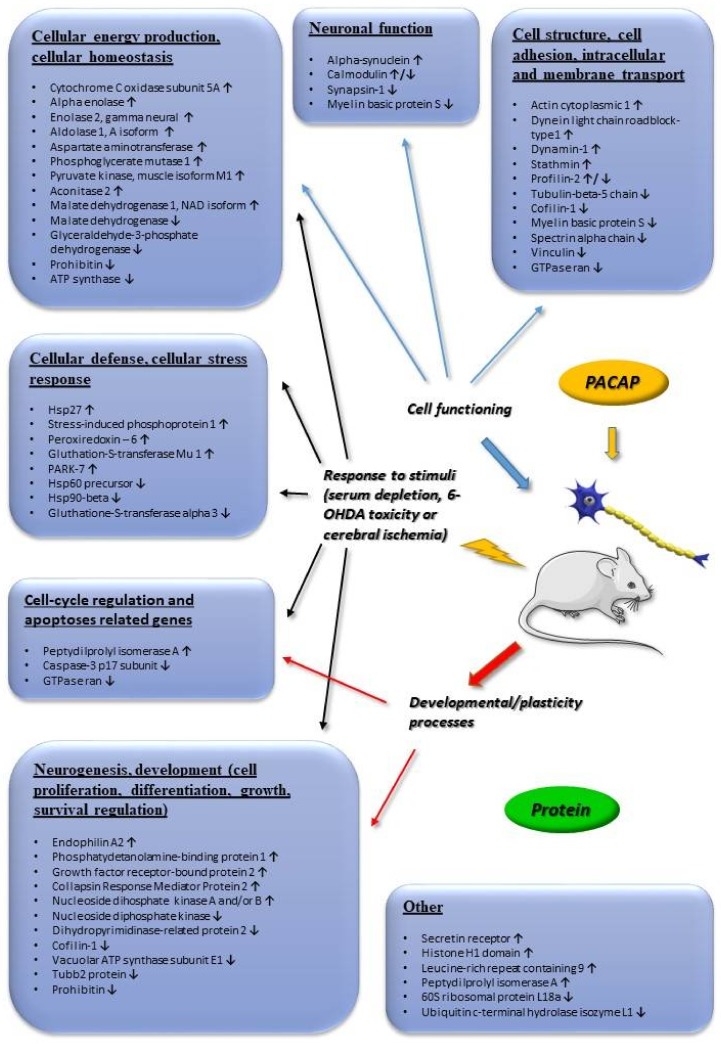
Main in vivo and in vitro proteomic changes induced by PACAP. Arrows indicate main up or downregulated genes. The data in the figure come from the references: [37,38,39,40,41,43,44,46]. Abbreviation: 6-OHDA (6-Hydroxydopamine).

Figure 1 summarizes transcriptomic data obtained from adrenal gland-derived cell lines, such as PC12 pheochromocytoma cells, mouse adrenal gland cells (MAG) and bovine chromaffin cells (BCC). We concentrate on changes that were found to be more than 2-fold. Figure 2 summarizes transcriptomic changes in vivo, while Figure 3 those of in vitro and in vivo proteomic studies. Obviously, it does not preclude smaller changes that can also be important during neuronal development and repair, but a complete list is beyond the scope of this present review. Transcriptomic and proteomic changes can be grouped according to several aspects. Focusing on neuronal development and neuroprotection, altered genes are involved in cellular defense, stress-related pathways, differentiation, apoptosis, cell cycle, angiogenesis and cellular homeostasis. The summarized changes are discussed in light of the known neurotrophic and neuroprotective effects of the peptide, and some marked changes with known neuronal functions are highlighted [32,33,34,35,36,37,38,39,40,41,42,43,44,45,46].

## 2. Involvement of PACAP in Neuronal Developmental Processes

PACAP has been described in the proliferation and differentiation of various neuronal cell lines, such as cortical neuroblasts [47], cerebellar granule cells [48,49], retinoblasts [50,51], sympathetic neuroblasts [52] and olfactory neuroblasts [53], detailed transcriptomic studies are available on adrenal cells. Data from chromaffin cells (PC12 pheochromocytoma cells) have been reviewed by Samal et al. [32], providing a thorough meta-analysis of PACAP-regulated genes.

A well-known PACAP-signaling target is the mitogen-activated protein kinase (MAPK) family with downstream CREB (cAMP response element binding protein) activation [24]. Transcriptomic data support this, as several Ras/MAP kinases and CREB-like proteins and modulators were strongly upregulated upon PACAP exposure (Figure 1). Another target of PACAP signaling is the calcium calmodulin pathway, members of which have also been shown to be upregulated upon PACAP exposure, as reported by in vitro and in vivo transcriptomic as well as proteomic studies (Figure 1, Figure 2 and Figure 3).

Several genes influenced by PACAP are involved in cell cycle regulation and cell proliferation. Transcriptomic data have revealed that PACAP upregulates members of the cell cycle regulator family cyclin (e.g., *cyclinD2*), and other complex factors that are part of the mitogenic signaling, such as *Jak1* (janus kinase 1) and *Stat3* (signal transducer and activator of transcription 3) [54,55] (Figure 1). These mitogenic effects are important in PACAP’s effects in neuronal proliferation, as it has been described in cortical neuroblasts [47], neural/glial progenitor cells [56], chicken neuroblasts [57] and cerebellar granule cells [58]. It seems that this function is dependent on receptor subtype expression and the neuronal differentiation stage during normal as well as tumorous development, since PACAP not only stimulates proliferation but can also be antimitotic and inhibit proliferation as it stimulates various signaling pathways depending on the receptor splice variant expression [52]. A receptor switch has been reported in cortical neuroblasts, determining the switch from mitogenic to anti-mitogenic actions depending on the neuronal differentiation stage [59]. A further modifying factor can be the tumorous nature of the blast cells, as opposite effects have been described in some malignant tumors of neuronal blast cells, such as medulloblastoma where PACAP inhibited proliferation [60] and in retinoblastoma, where decreased cell viability was observed upon PACAP treatment [61].

During brain development, PACAP is involved not only in the proliferation, but also in migration and differentiation of developing neuronal cells. Numerous transcription factors involved in neuronal differentiation are also upregulated by PACAP. Among them, *transforming growth factor beta (TGFbeta)*, *nerve growth factor-induced protein A (Egr1)*, *brain derived neurotrophic factor (BDNF), fibroblast growth factor (FGF21*), *insulin-like growth factor (Igf1)* and *bone morphogenic protein receptor type 2* are very important factors during brain development [62,63,64,65,66,67] (Figure 1 and Figure 2). In neuronal differentiation, PACAP is known to be involved in axonal growth cone development and axonal elongation [68], in neuronal migration [69,70] and in neuronal patterning [71]. Transcriptomic data have revealed, for example, an upregulation of *plexin A2* (Figure 1), which is important in axon elongation [72]. PACAP also influences synaptogenesis, possibly via the upregulated *SNAP-25/SNARE complex (soluble NSF(N-ethylmaleimide-sensitive factor attachment protein, and its receptor)* [73] and *synaptostagmin 4* [74] in addition to *AMPA receptor 1 (α-amino-3-hydroxy-5-methyl-4-isoxazolepropionic acid)* (Figure 1). Neurofilament polypeptide and *calponin 3*, parts of the neuronal cytoskeleton [75,76], are also upregulated by PACAP (Figure 1). One of the genes showing most marked changes is *tenascin C* (via *annexin-2*) (Figure 1 and Figure 2), which is known to form a net around neurons during development and also in adulthood. The PACAP-induced elevation in *tenascin C* and *annexin-2* may indicate a significant involvement of PACAP in neuronal network building [77,78].

Numerous experimental data show that PACAP is involved in the normal development of the brain. Most data are available from the cerebellum, where PACAP regulates cell migration, proliferation and differentiation of developing granular cells [79]. Layer-specific signaling has been mapped for PACAP during the development of cerebellar neurons [80]. PACAP is an important stop signal during the migration of cells from the external to internal granule layer [79]. The involvement of PACAP in cerebellar development has been studied not only in rodents, but also in monkeys [81]. During development, well-orchestrated programmed cell death plays a major role in the final cell composition of the brain. One of the most intensively studied effects of PACAP is apoptosis. PACAP influences several genes involved in apoptosis, resulting in numerous changes in the apoptotic signaling, initiation and execution. Among others, *14-3-3-ζ,* upregulated by PACAP, negatively regulates apoptosis by binding and inactivating the pro-apoptotic bad and bax proteins [82] (Figure 2 and Figure 3). Transcriptomic studies have also shown the down-regulation of caspase and bad by PACAP, also confirmed by several proteomic studies (Figure 2 and Figure 3). A large amount of data shows the influence of PACAP on apoptosis during cerebellar development under normal circumstances [83]. Several further transcriptomic and proteomic changes prove the involvement of PACAP in neuronal development that is evidenced from experimental data in cell lines and from in vivo animal studies. Furthermore, data indicate a significant involvement of PACAP in neuronal network building. As PACAP is an important regulator of neuronal development, it is not surprising that several developmental malformations have been attributed to dysregulation of PACAP-mediated signaling. Disturbance of PACAP expression in PACAP gene deficient mice has been associated with irregular dendritic arborization [84], disturbed cerebellar development [85], alterations in ectomesenchymal differentiation in facial development [86,87].

PACAP also influences development of glial cells. PACAP stimulates the proliferation of oligodendrocytes but delays their maturation [88], and so it is part of the myelination processes in the central nervous system, further confirmed by the upregulated *plexin A2* gene (Figure 1), which is involved in myelination processes [89]. PACAP also affects astrogliogenesis and differentiation [14,90]. Interestingly, while PACAP promotes differentiation of astroglial cells under normal developmental circumstances [58], it inhibits proliferation of glioblastoma cells [91]. The third major glial cell type is microglia, the origin of which is mesodermal, in contrast to the neuroectodermal origin of all other cells in the central nervous system. In spite of this different origin, PACAP seems to also play a role in the differentiation and function of microglial cells [92].

## 3. Neuroprotective Effects of PACAP

Transcriptomic and proteomic studies also show that PACAP is an important regulator of normal neuronal functioning, even after development. It functions as a neurotransmitter and neuromodulator [34] and affects glial functions [14]. PACAP affects several physiological actions in the adult brain and is involved in complex behavioral processes, such as feeding, motor functions and thermoregulation [2,3,5,24]. PACAP is also involved in cognitive performance, fear and anxiety [12,14]. Not surprisingly, adult PACAP knockout mice display several abnormalities. The main shortcoming of using knockout mice is that the lack of PACAP induces several compensatory pathways, which can partially or totally compensate for the lack of the neuropeptide [93] and so data might be misleading. Lack of a symptom in the knockout mice does not necessarily mean that the peptide is not involved in this process under normal circumstances, when compensatory pathways are not involved. In contrast, when an alteration is found in the gene deficient mice, it makes it probable that the peptide plays a significant role in the regulation of that process. Although the morphological differences in PACAP gene deficient mice seem to be subtle, several functional disturbances have been described in mice lacking endogenous PACAP [19,94]. These alterations could be caused by biochemical and cell signaling differences due to the lack of endogenous PACAP expression [94,95]. For example, altered stress-coping reactions have been described in PACAP heterozygous animals [96,97], altered psychomotor behavior [98] and decreased cognitive functions [99].

Of special importance is the observation that PACAP-regulated pathways are essential in neuroprotective processes as part of the endogenous protective machinery as a cell stress-responsive neuropeptide. This is evidenced by both in vitro and in vivo observations. In vitro, PACAP upregulates several important genes and proteins involved in cellular protection against various harmful stimuli. For example, PACAP has been shown to be protective against toxicity induced by several substances, such as ethanol, nicotine [23], ceramide [69,100], 6-hydroxydopamine [101], the HIV envelope protein [102] and cisplatin [103]. The strong neuroprotective property of PACAP can also be observed in vivo in several different models of neuronal injury. Among others, neuroprotective actions have been shown in excitotoxic injuries [104], 6-hydroxydopamine- [25], β-amyloid- [105] and ischemia-induced injuries [106]. The in vivo neuroprotective action of PACAP can be observed when the peptide is given exogenously and also in knockout mice reflecting the endogenous function of the peptide. Given the importance of PACAP in neuronal protection and repair, it is not surprising that mice lacking endogenous PACAP are more vulnerable to different stressors and injuries [19]. The increased sensitivity of PACAP knockout mice has been demonstrated in cerebral and retinal ischemia [20,22], in delayed axonal regeneration [107] and in paraquat-induced model of Parkinson’s disease [108].

PACAP has long been known as a potent in vivo neuroprotective peptide when administered in a variety of neuronal lesions [16]. PACAP was first described as protecting hippocampal neurons from death in a model of global cerebral ischemia [109], later followed by studies showing the neuroprotective efficacy of PACAP in focal cerebral ischemia induced by permanent or transient middle cerebral artery occlusion [110,111]. Importantly, the peptide is neuroprotective even if the administration follows 4 h after the induction of stroke [110], indicating a relatively wide therapeutic window for PACAP’s actions. These studies have later been confirmed by several research groups, in both rats and mice. Ohtaki and coworkers have shown that the increased infarct size in PACAP knockout mice can be compensated by exogenous PACAP [20]. The neuroprotective effects of PACAP have been described not only in models of ischemic injuries, but also in neurodegenerative diseases [17], such as models of Parkinson’s disease [25,112], striatal model of Huntington’s disease [113] or spinobulbar muscular atrophy [114] and also in traumatic brain injury [115]. Gasperini and colleagues have studied the proteome of diverse brain areas after PACAP injection [39] (Figure 3). They found that PACAP is involved in assorted molecular processes that may also be important during neuronal protection and regeneration, such as synaptic plasticity, cellular differentiation, proteins of apoptotic cascades and stress pathways.

Investigating the in vivo neuroprotective action of PACAP, transcriptomic and proteomic studies have mainly focused on ischemic injuries. Hori and coworkers have analyzed in detail the alterations after middle cerebral artery occlusion-induced cerebral ischemia [37,116] (Figure 2 and Figure 3). They have described several changes that can be additive factors to exert protective effects in cerebral ischemic injuries. Dejda and colleagues [36] have analyzed a number of genes associated with the actions of PACAP, mainly involved in apoptosis and inflammation regulation, cell signaling and edema formation (Figure 2). Chen and coworkers have detected a number of genes altered in the early and late phases of cerebral infarct, using the same rat model [117] (Figure 2). They found that more genes were upregulated in the later phase of post-cerebral artery occlusion, than in the early phase, suggesting a more important protective role of PACAP in later phases of cerebral ischemia. Several of the PACAP-responsive transcripts have roles in neuronal processes, neuronal repair and plasticity. In addition to the developmental factors mentioned above, *ninjurin*, which is involved in axonal repair [118], has been shown to be upregulated after PACAP treatment (Figure 1). Brifault and coworkers [119] have analyzed the transcriptome after middle cerebral artery occlusion and PACAP-producing stem cell transplantation into the ventricular system. Their analysis showed that PACAP is involved in several regulatory pathways, such as chemotactic and inflammatory networks, and this may induce the phenotypic change in the microglial population towards neuroprotection [119]. Another research group has analyzed ischemic core and penumbra areas separately by transcriptomic and proteomic approaches [37,100]. They have identified several groups of genes and proteins to be differentially altered after the induction of cerebral ischemia. Altogether, transcriptomic and proteomic data confirm the involvement of anti-apoptotic and anti-inflammatory pathways in the PACAP-induced reduction of lesion size and post-injury repair and regeneration. For example, heat shock proteins and peroxiredoxin, with important functions in cellular stress-response [120,121], are upregulated by PACAP, as well as protective factors PARK7 and gluthathion *S*-transferase [122,123] (Figure 1, Figure 2 and Figure 3). Changes in factors involved in apoptotic pathways, inflammation, cellular defense and metabolism have been described by all these above-mentioned studies, with some differences probably due to different animal strain/source as well as the different time-window after the insult or the exact brain region analyzed. For example, Chen and co-workers analyzed brain samples 1 and 24 h after stroke, while Hori and co-workers 6 and 24 h after the induction of ischemia [37,117]. In contrast, Brifault and colleagues performed transcriptomic analysis 7 and 14 days after stroke [119]. Acute changes can be represented by 1 h data, samples obtained 6 h after stroke induction already a sub-acute phase, while data obtained 7 or 14 days after stroke already reflect regenerative changes. From minutes to weeks after the ischemic event, different processes play a major role in the ischemic cascade, like neuroprotection, inflammation followed by tissue repair and plasticity, naturally reflected also in the transcriptomic and proteomic changes [124]. In addition, numerous transcriptomic differences have been revealed between ischemic core and penumbra regions, the size of which gradually changes over time, further influencing the time-course and region-specific transcriptomic and proteomic alterations. Therefore, direct comparison of data obtained by different research groups using different experimental paradigms is not possible, but their main findings point to the involvement of PACAP in both acute and chronic responses after an ischemic insult and support the neuroprotective efficacy of the peptide.

In conclusion, the long-known neuroprotective effects of PACAP are supported by numerous transcriptomic and proteomic data, further indicating the involvement of PACAP in synaptic plasticity, neuronal growth and differentiation, apoptosis and inflammation and axonal growth. These data shed more light on the neuroprotective action of the peptide shown in several different models of neuronal injuries. Recent results indicate that this effect of PACAP is not only present in animal models but is also implicated in human diseases, in acute and chronic neuronal pathological conditions, and endogenous decline of the peptide can indicate neuronal degeneration [11]. Due to its human relevance, the therapeutic and/or diagnostic value of PACAP is gaining more importance, and thus, more detailed mechanistic approach is necessary in various neuronal injury models. The strong evidence for the neuroprotective effects of PACAP makes it a good candidate for future drug development in ischemic conditions and neurodegenerative diseases. Interestingly, however, closest to the therapeutic use is an antibody against PAC1 receptor that is already under clinical trial for treatment of migraine attacks, based on the effect of PACAP via PAC1 receptor in triggering migraine-type headaches [125]. Yet, this can lead to the blockade of various other, beneficial pathways induced by PACAP, as outlined in the present review. This calls for the necessity of further transcriptomic and proteomic studies that can help to reveal possible effects of future PACAP-based treatments as well as side-effects of blocking PACAP-signaling.
